# Large mandibular mass with several floating teeth: granular cell ameloblastoma

**DOI:** 10.1093/jscr/rjad666

**Published:** 2023-12-14

**Authors:** Samuel E Razmi, Richard E Hayden, Brent A Chang

**Affiliations:** Texas A&M School of Medicine – ENMED, 1020 Holcombe Boulevard, Houston, TX 77030, United States; Department of Otorhinolaryngology – Head and Neck Surgery, Mayo Clinic Arizona, 5777 E Mayo Boulevard, Phoenix, AZ 85054, United States; Department of Otorhinolaryngology – Head and Neck Surgery, Mayo Clinic Arizona, 5777 E Mayo Boulevard, Phoenix, AZ 85054, United States

**Keywords:** ameloblastoma, mandibular mass, granular cell ameloblastoma

## Abstract

Ameloblastomas are benign, locally aggressive, odontogenic epithelial neoplasms. We present a patient with a rare granular cell ameloblastoma. This is a case report and literature review conducted from July 2022 to the present. Our 52-year-old male patient presented to the clinic with a several month history of right lower lateral lip swelling and dental complaints. On imaging and physical exam, we noticed a large heterogenous, multiloculated, expansile lesion of the right mandible with several floating teeth. After incisional biopsy confirmed multicystic granular cell ameloblastoma, the patient underwent successful surgical resection and osteocutaneous fibula free-flap reconstruction. At the time of writing this report, 7 years after resection, the patient is alive with no evidence of recurrence. The recognition and treatment of mandibular lesions can represent significant clinical challenges, especially for rarely seen subtypes such as the granular cell ameloblastoma. Special consideration must be given for the identification and treatment of these neoplasms.

## Introduction

Mandibular lesions can be of odontogenic or nonodontogenic origin. Ranging from benign cysts to malignant solid tumors, there is a wide spectrum of potential diagnoses when facing a mandibular mass in the clinic. These lesions are often difficult to diagnose and require a multidisciplinary approach in treatment and diagnosis. Mandibular lesions are rare themselves, and ameloblastoma is no exception to this, being one of the more infrequent diagnoses of mandibular lesions. Ameloblastomas have several subtypes and classifications, with multicystic granular cell ameloblastoma being one of the rarer presentations reported in the literature, comprising less than 5% of all ameloblastomas [1].

## Case presentation

**Figure 1:**
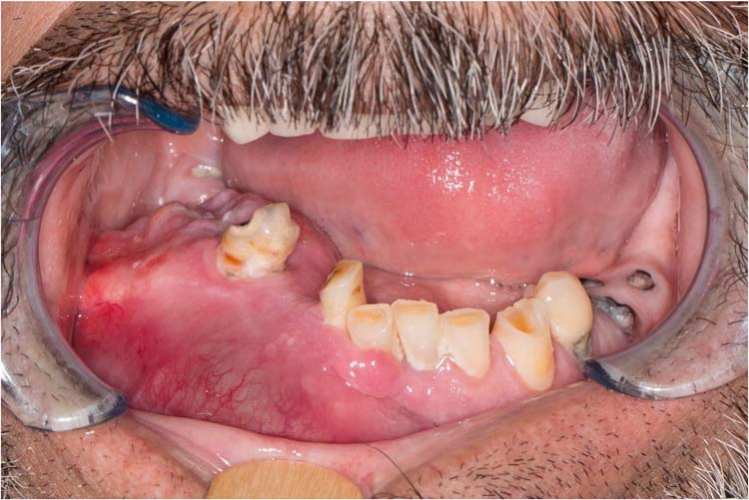
Physical examination revealing an evident large right mandibular lesion present in the patient.

**Figure 2:**
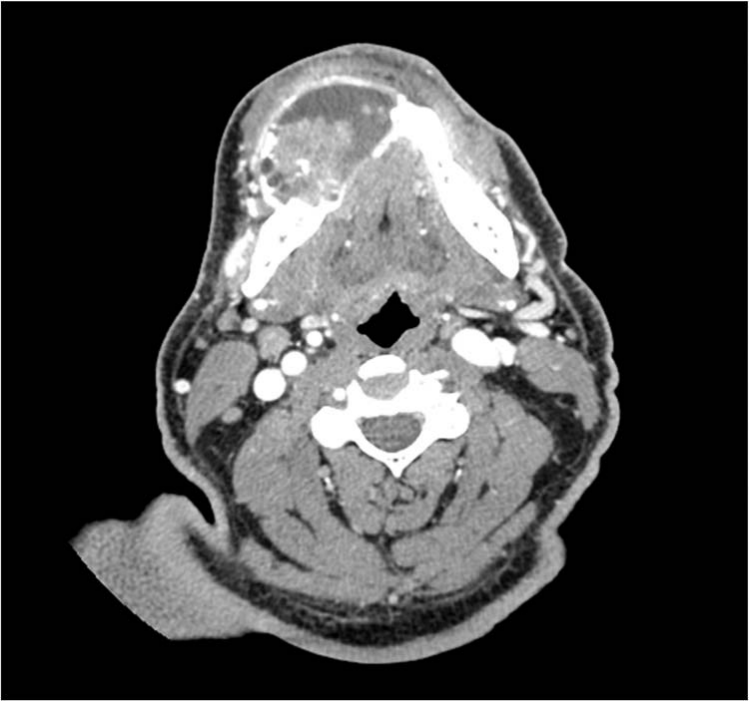
CT imaging demonstrating the heterogeneous, multiloculated, expansile lesion stretching in the buccal-lingual direction.

**Figure 3:**
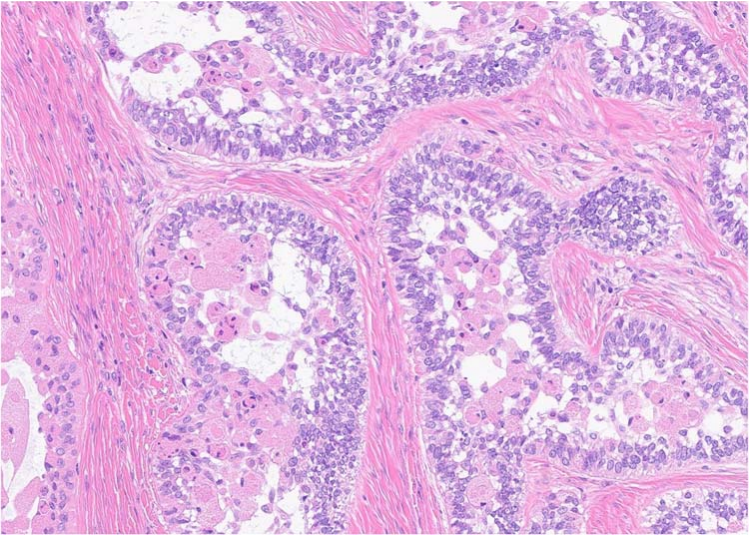
Histopathology (400×, H&E strain) demonstrating characteristic granular cell ameloblastoma features such as epithelial tumor islands with columnar cells exhibiting peripheral palisading, stellate reticulum-like areas, granular cells, and reverse polarization.

A 52-year-old male presented with a 4-month history of right lower lateral lip swelling and dental complaints. On physical examination, there was a large right mandibular mass with several floating teeth extending from the symphysis to the angle (Fig. 1). The patient’s history was notable for hypertension and a 36-pack year smoking history. He denied any other concerning symptoms. Computed tomography (CT, Fig. 2) imaging demonstrated at large heterogeneous, multiloculated, expansile lesion of the right mandible. An incisional biopsy was performed, and results demonstrated epithelial tumor islands with columnar cells exhibiting peripheral palisading, stellate reticulum-like areas, granular cells, and reverse polarization (Fig. 3). Following diagnostic confirmation of multicystic granular cell ameloblastoma, the patient underwent successful surgical resection and reconstruction. Final pathology showed a 5.0-cm granular type conventional multicystic ameloblastoma with negative surgical margins. At time of report, 7 years after resection, our patients is alive with no evidence of recurrence.

## Discussion

Ameloblastoma is a rare, benign, and locally aggressive odontogenic neoplasm of epithelial origin that typically presents near the ramus or mandibular molars. They are mostly of the conventional multicystic subtype but can also less frequently present as unicystic, extraosseous, or metastasizing subtypes. Other than histopathological differences, the multicystic granular cell ameloblastoma does not have different treatment or prognosis than a conventional multicystic ameloblastoma. Affecting all genders equally, granular cell ameloblastomas typically present in the fourth decade of life [2]. Commonly, they present with swelling and pain but may occasionally cause ulcerations or mobility of teeth. As with our patient, these teeth are sometimes described as floating when the alveolar bone destruction of the roots give the teeth a floating appearance on imaging.

On CT, multicystic granular cell ameloblastomas present as a multilocular radiolucent destruction of bone, appearing as a honeycomb or soap bubble appearance. Anatomically, they are not confined to the alveolar bone and routinely perforate cortical bone. These findings are not pathognomonic, and thus, other odontogenic diagnoses, such as odontogenic keratocyte and central giant cell granuloma, must be considered as well [3]. Radiologically, granular cell ameloblastomas typically expand in the buccal-lingual direction, which can be seen in our patient (Fig. 2). While helpful, radiology is only supplemental in determining a final diagnosis.

Pathological analysis serves as the basis for final diagnosis of these odontogenic tumors. There are at least six histopathological patterns associated with conventional multicystic ameloblastoma. The most common is of the conventional multilocular type with a follicular histological pattern, which is consistent islands of odontogenic epithelium in fibrous connective tissue, classic peripheral palisading, and stellate reticulum-like areas. Our patient was of the rarer granular type with a presentation consistent with stellate reticulum-like cells having large eosinophilic rounded or polyhedral granular cells. Interestingly, microscopic pattern has no documented prognostic significance [4].

The treatment of choice for large multicystic granular cell ameloblastoma is segmental resection followed by the reconstruction of the defect. Current recommendations indicate a 1–2 cm margin minimum for segmental resections [5]. For smaller unicystic ameloblastomas, enucleation and curettage can serve as more conservative forms of treatment. Radiation therapy may be required for inoperable patients. Our patient underwent right segmental mandibulectomy from angle to left canine followed by left osteocutaneous fibula free-flap reconstruction of right mandibulectomy. Granular cell ameloblastomas have a higher rate of recurrence when compared against conventional multicystic ameloblastomas, and thus, a follow-up of at least 10 years should be considered in these patients [2]. At time of report, 7 years after resection, our patients is alive with no evidence of recurrence.

## Conclusion

The granular cell ameloblastoma is a rare variant of an already uncommon mandibular lesion. While the neoplasm has unique histopathological findings, the prognosis and treatment of these lesions do not differ greatly from conventional ameloblastomas. The recognition and treatment of these lesions can represent significant clinical challenges, and as such, a better understanding of their composition can provide therapeutic benefits.
